# Antimicrobial peptides and other potential biomarkers of critical illness in SARS‐CoV‐2 patients with acute kidney injury. AMPAKI‐CoV study

**DOI:** 10.14814/phy2.15945

**Published:** 2024-02-08

**Authors:** Lucas Ferreira Theotonio dos Santos, Hermes Vieira Barbeiro, Denise Frediani Barbeiro, Heraldo Possolo de Souza, Fabiano Pinheiro da Silva

**Affiliations:** ^1^ Laboratório de Emergências Clínicas, Faculdade de Medicina Universidade de São Paulo São Paulo Brasil

**Keywords:** acute kidney injury, cathelicidins, COVID‐19, defensins, innate immunity, Interleukin‐10

## Abstract

Antimicrobial peptides (AMPs) constitute a complex network of 10–100 amino acid sequence molecules widely distributed in nature. While over 300 AMPs have been described in mammals, cathelicidins and defensins remain the most extensively studied. Some publications have explored the role of AMPs in COVID‐19, but these findings are preliminary, and in vivo studies are still lacking. In this study, we report the plasma levels of five AMPs (LL‐37, α‐defensin 1, α‐defensin 3, β‐defensin 1, and β‐defensin 3), using the ELISA technique (MyBioSource, San Diego, CA, United States, kits MBS2601339 (beta‐defensin 1), MBS2602513 (beta‐defensin 3), MBS703879 (alpha‐defensin 1), MBS706289 (alpha‐defensin 3), MBS7234921 (LL37)), and the measurement of six cytokines (tumor necrosis factor‐α, interleukin‐1β, interleukin‐6, interleukin‐10, interferon‐γ, and monocyte chemoattractant protein‐1), through the magnetic bead immunoassay Milliplex® and the MAGPIX® System (MilliporeSigma, Darmstadt, Germany, kit HCYTOMAG‐60 K (cytokines)), in 15 healthy volunteers, 36 COVID‐19 patients without Acute Kidney Injury (AKI) and 17 COVID‐19 patients with AKI. We found increased levels of α‐defensin 1, α‐defensin 3 and β‐defensin 3, in our COVID‐19 population, when compared to healthy controls, along with higher levels of interleukin‐6, interleukin‐10, interferon‐γ, and monocyte chemoattractant protein‐1. These findings suggest that these AMPs and cytokines may play a crucial role in the systemic inflammatory response and tissue damage characterizing severe COVID‐19. The levels of α‐defensin 1 and α‐defensin 3 were significantly higher in COVID‐19 AKI group in comparison to the non‐AKI group. Furthermore, IL‐10 and the product IL‐10 × IL‐1B showed excellent performance in discriminating AKI, with AUCs of 0.86 and 0.88, respectively. Among patients with COVID‐19, AMPs may play a key role in the inflammation process and disease progression. Additionally, α‐defensin 1 and α‐defensin 3 may mediate the AKI process in these patients, representing an opportunity for further research and potential therapeutic alternatives in the future.

## Graphical abstract

The activation of antimicrobial peptides is induced by the infiltration of SARS‐CoV‐2 into the epithelial cells. Alpha‐defensins exhibit a positive correlation with renal injury, whereas beta‐defensin 3 is associated with pulmonary impairment. The question of whether these peptides exert a causal influence or serve as modulators of the pathological pathways remains contentious. Moreover, the ensuing immune reaction escalates the concentrations of interleukin‐10 (IL‐10).

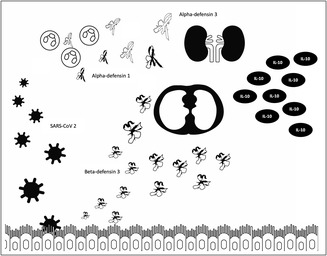

## 1 INTRODUCTION

Antimicrobial peptides (AMPs), also known as host defense peptides (HDP), constitute a diverse group of molecules present in both eukaryotes and prokaryotes. Initially investigated for their direct antimicrobial properties, recent studies increasingly highlight their role as major regulators of the immune response (Pinheiro Da Silva & Machado, 2012). In higher organisms, cathelicidins, α‐, and β‐defensins are the most widely investigated AMPs.

Humans and rodents each possess one cathelicidin family member, produced by immune and epithelial cells and named LL‐37 and CRAMP (cathelin‐related antimicrobial peptide), respectively. Defensins, on the other hand, are divided in three main classes in mammals: the α‐, β‐, and θ‐defensins. Human α‐defensins 1–4 are primarily present in neutrophils, whereas human β‐defensins, like cathelicidins, are produced by several types of immune and epithelial cells.

COVID‐19 (Coronavirus Disease 2019) is an infectious disease caused by severe acute respiratory syndrome coronavirus 2 (SARS‐CoV‐2). It was first described in China in 2019 and gained global attention, since it rapidly became a devastating pandemic. Worldwide, around 6.9 million people already died because of this disease, which has a diverse clinical course, but generally includes signs and symptoms of systemic inflammation. Although the lung is the most affected organ, SARS‐CoV‐2 severe infections often lead to Acute Kidney Injury (AKI) (Kellum et al., 2020; Nadim et al., 2020). The mechanisms of kidney damage still need to be fully elucidated, but a combination of direct viral damage to the renal cells, systemic inflammation and endothelial dysfunction have been implicated (Armaly et al., 2021; Stasi et al., 2020). AKI is more common in hospitalized COVID‐19 patients, being a marker of worse prognosis and increased mortality (Ng et al., 2020; Qian et al., 2021).

Antimicrobial peptides have received limited attention in COVID‐19 research. A recent study demonstrated in vitro that LL‐37 not only binds to SARS‐CoV‐2 spike 1 protein, but also cloaks the ligand‐binding domain of angiotensin‐converting enzyme 2 (ACE2), protecting against virus entry into the cell (Wang et al., 2021). In addition, there are reports that α‐defensins 2 and 5 also protect epithelial cells in vitro from SARS‐CoV‐2 invasion, using an analogous mechanism (Wang et al., 2020; Zhang et al., 2021). However, for a better comprehension of the role of AMPs in severe COVID‐19, in vivo studies are needed.

In this study, we report the plasma levels of 5 antimicrobial peptides (LL‐37, α‐defensin 1, α‐defensin 3, β‐defensin 1, and β‐defensin 3) in 15 healthy subjects, 36 COVID‐19 patients without AKI and 17 COVID‐19 patients with AKI. We also measured several cytokines (tumor necrosis factor‐α, interleukin‐1β, interleukin‐6, interleukin‐10, interferon‐γ, and monocyte chemoattractant protein‐1). These AMPs and cytokines were chosen because they have been implicated in several other systemic inflammatory conditions (Cheng et al., 2015; Pinheiro Da Silva & Machado, 2017; Qi et al., 2016). Interestingly, HBD‐1 is expressed in kidney epithelial cells, among others, and HBD‐3 is produced by lung epithelial cells (Donovan & Topley, 2003; Lehmann et al., 2002; Schnapp et al., 1998). In situ hybridization localized the HBD‐1 mRNA in the epithelial layers of the loops of Henle, distal tubules, and the collecting ducts of the kidney (Ganz & Lehrer, 1997). Given that finding, one may suppose that HBD‐1 is a potential biomarker of tubular damage. Overall, all these antimicrobial peptides are potential biomarkers of disease activity, organ damage and risk of death.

## 2 METHODS

### 2.1 Selection of patients and samples collection

This study received approval from the Ethics Committee of our hospital (protocol # 30417520.0.0000.0068). A written informed consent was obtained from the patient or responsible family member for patient inclusion, in the hospital admission. For healthy individuals, the written consent was obtained in the moment of the sample collection.

**FIGURE 1:**
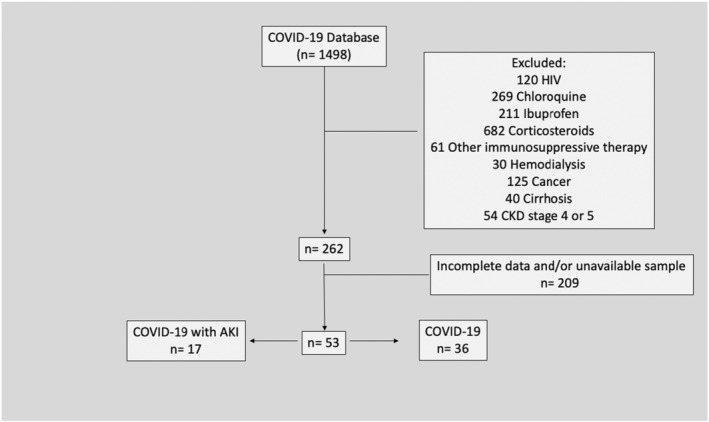
Selection of patients according to exclusion criteria.

We included a total of 53 patients from a database of 1498, applying exclusion criteria (see Figure 1). They were divided in two groups, according to KDIGO criteria (Khwaja, 2012): those without AKI (COVID‐19 group, *n* = 36) and those with AKI (COVID‐19 AKI group, *n* = 17). Admission creatinine levels served as the baseline. We also included a control group consisting of 15 healthy volunteers.

Patients aged 50 years and older, with COVID‐19 diagnosis confirmed by real‐time polymerase chain reaction (RT‐PCR) and at least 2 serum creatinine values during in‐hospital stay were included. Healthy individuals aged 50 years and older, without symptoms of COVID‐19 in the prior 30 days and without exclusion criteria were selected.

Blood samples were collected within 24 h of hospital admission, with plasma immediately separated by centrifugation and stored at −80°C for subsequent analysis.

### 2.2 Measurement of antimicrobial peptides and cytokines

Plasma levels of α‐defensins 1 and 3, β‐defensins 1 and 3, and LL‐37 were measured using a kit based on the ELISA technique (MyBioSource, San Diego, CA, United States, kits MBS2601339 (beta‐defensin 1), MBS2602513 (beta‐defensin 3), MBS703879 (alpha‐defensin 1), MBS706289 (alpha‐defensin 3), MBS7234921 (LL37)). Tumor necrosis factor‐α (TNF‐α), interleukin‐1β (IL‐1 β), interleukin‐6 (IL‐6), interleukin‐10 (IL‐10), interferon‐γ (IFN‐γ), and monocyte chemoattractant protein‐1 (MCP‐1) plasma levels were measured using the magnetic bead immunoassay Milliplex® and the MAGPIX® System (MilliporeSigma, Darmstadt, Germany, kit HCYTOMAG‐60K (cytokines)).

### 2.3 Statistical analysis

Continuous clinical characteristics and laboratory parameters were analyzed using the Kruskal–Wallis test or the Mann–Whitney test, as appropriate. The Chi‐Square test of independence was used for categorical clinical values. Results were presented as medians and interquartile ranges for continuous variables and medians and percentages for categorical values. Plasma measurements were analyzed using the Kruskal–Wallis test, followed by the Mann–Whitney *U* test for post hoc analysis. ROC curve analysis was performed to assess the ability of cytokines and antimicrobial peptides to discriminates between AKI and non‐AKI patients. Correlation was assessed using the Pearson correlation test, with a coefficient (r) of 0.5 or greater, or −0.5 or less, considered significant. All analyses were conducted using R statistical software (www.r‐project.org). A *p*‐value ≤ 0.05 was considered significant.

## 3 RESULTS

There were no significant differences in age, gender, or comorbidity among the study groups, indicating a similar profile among individuals (see Table 1). The COVID‐19 AKI group, however, exhibited longer hospital stays, higher rate of Intensive Care Unit (ICU) admission, more days in mechanical ventilation and higher mortality (Table 1). Stratified by KDIGO guidelines creatinine criteria (Khwaja, 2012), we had four KDIGO 3, four KDIGO 2 and nine KDIGO 1 AKI individuals. No data about diuresis were consistent available so a more reliable stratification was not possible. Table 2 displays values for several laboratory parameters obtained from our COVID‐19 patients within 24 h hospital admission. Blood gases, urea, creatinine, electrolytes, blood cells count, C‐reactive protein, and D‐dimer values were investigated. Interestingly, we could detect no statistical differences other than in peak creatinine, when the COVID‐19 and the COVID‐19 AKI groups were compared, putting in evidence that these parameters are poor indicators of inflammatory state, disease severity or risk of death in this situation (AKI vs non‐AKI).

**TABLE 1:** Clinical characteristics of the healthy individuals (*n* = 15) and patients included in our study (COVID‐19 group = 36 patients; COVID‐19 AKI group = 17 patients).

Clinical data	Control group	COVID‐19	COVID 19 + AKI	*p*‐Value
	Median (IQR) or *n* (%)	Median (IQR) or *n* (%)	Median (IQR) or *n* (%)	
Age (years)	63 (57–75)	64.5 (51–82)	64 (53–88)	0.816
Gender (male)	9 (60%)	25 (69.4%)	12 (70.6%)	0.771
Comorbidities	–	–	–	–
Hypertension	7 (46.7%)	22 (61.1%)	12 (70.6%)	0.382
Diabetes	6 (40%)	15 (41.7%)	8 (47.1%)	0.908
Heart failure	1 (6.7%)	1 (2.8%)	2 (11.8%)	0.426
Coronary artery disease	2 (13.3%)	3 (8.3%)	2 (11.8%)%	0.844
Asthma or COPD	1 (6.7%)	2 (5.6%)	1 (5.9%)	0.988
Obesity	3 (20%)	6 (16.7%)	2 (11.8%)	0.814
Symptoms duration prior to admission (Days)	–	9.5 (1–15)	7 (2–13)	0.351
Mechanical ventilation	–	2 (5.6%)	8 (47.1%)	**<0.001**
Lenght of stay (Days)	–	13.5 (7–90)	20 (7–56)	**0.005**
ICU admission	–	16 (44.4%)	16 (94.1%)	**0.001**
Death	–	4 (11.1%)	11 (64.7%)	**<0.001**

*Note*: Mann–Whitney test was used to compare symptoms duration, days in mechanical ventilation, length of stay, rates of ICU admission and mortality of the COVID groups. The Kruskal–Wallis test was used to analyze the other variables in the three study groups (IQR = interquartile range). Bold values indicate significant of *p* values.

**TABLE 2:** Laboratory parameters of the patients included in our study (COVID‐19 group = 36 patients; COVID‐19 AKI group = 17 patients).

Laboratory	COVID‐19		COVID‐19 + AKI		*p*‐Value	Normal values
	Median	IQR	Median	IQR		
Gasometry	–	–	–	–	–	–
pH	7.44	0.05	7.41	0.14	0.196	7.45–7.35
pO2	66.1	21	68.3	24.73	0.283	100–80 mmHg
pCO2	36	8.2	40.75	19.13	0.449	45–35 mmHg
Bicarbonate	24.8	4.7	23.8	4.82	0.406	28–21 mmol/L
Base Excess	1.6	3.5	−1.5	4.7	0.164	3 to −3 mmol/L
Urea	40	15	40	25	0.561	50–10 mg/dL
Phosphate	2.7	0.9	3.15	0.75	0.195	4.5–2.7 mg/dL
Sodium	140	4	140	4.5	0.624	145–135 mEq/L
Magnesium	2.12	0.27	2.11	0.29	0.575	2.55–1.58 mg/dL
Potassium	4.05	1.05	4.1	0.8	0.894	5.0–3.5 mEq/L
Chloride	103	9	103	5.5	0.368	107–98 mEq/L
Calcium (ionic)	4.6	0.34	4.67	0.32	0.109	5.29–4.49 mg/dL
Creatinine (on admission)	0.91	0.21	0.93	0.37	0.849	0.7–1.5 mg/dL
Creatinine (peak)	0.95	0.205	1.93	1.23	<0,001	0.7–1.5 mg/dL
Leukocytes	7925	3170	8010	7690	0.985	11,000–4000 n/mm^3^
Neutrophils	5960	2898.75	7320	5955	0.717	7500–2500 n/mm^3^
Eosinophils	0	57.2	0	10	0.094	440–40 n/mm^3^
Basophils	15	10	10	20	0.377	100–0 n/mm^3^
Lymphocytes	975	760	720	485	0.075	3500–1500 n/mm^3^
Monocytes	530	310	380	235	0.153	800–200 n/mm^3^
CRP	172.8	94.2	155.9	118.7	0.435	<5 mg/L
D‐Dímer	1143	1606	4306	24,359	0.184	<500 ng/mL

*Note*: Mann–Whitney test was used to compare the COVID groups, normal values are exhibited only for reference (IQR = interquartile range).

**FIGURE 2:**
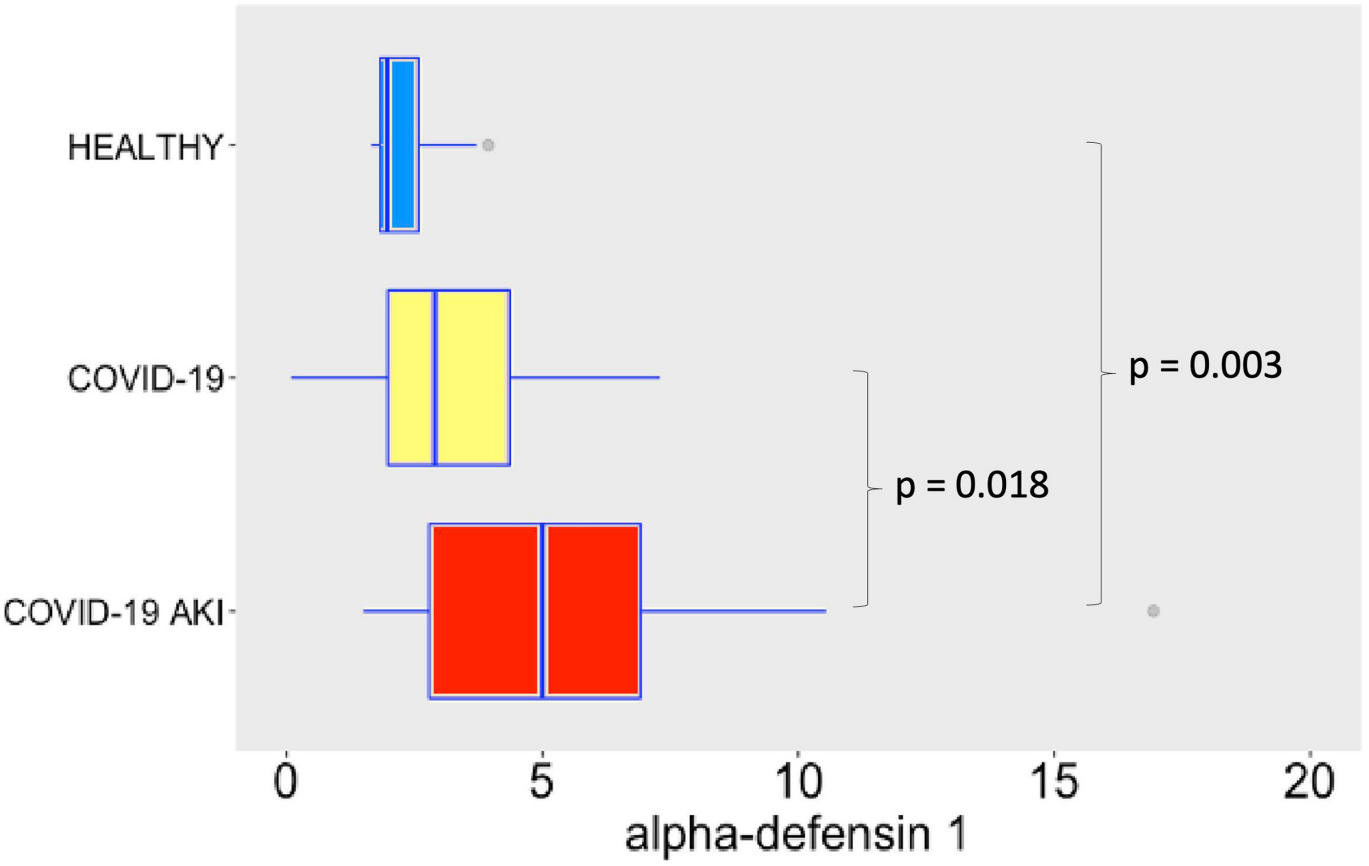
Plasma levels of α‐defensin 1 in ng/mL. The dots represent outliers.

**FIGURE 3:**
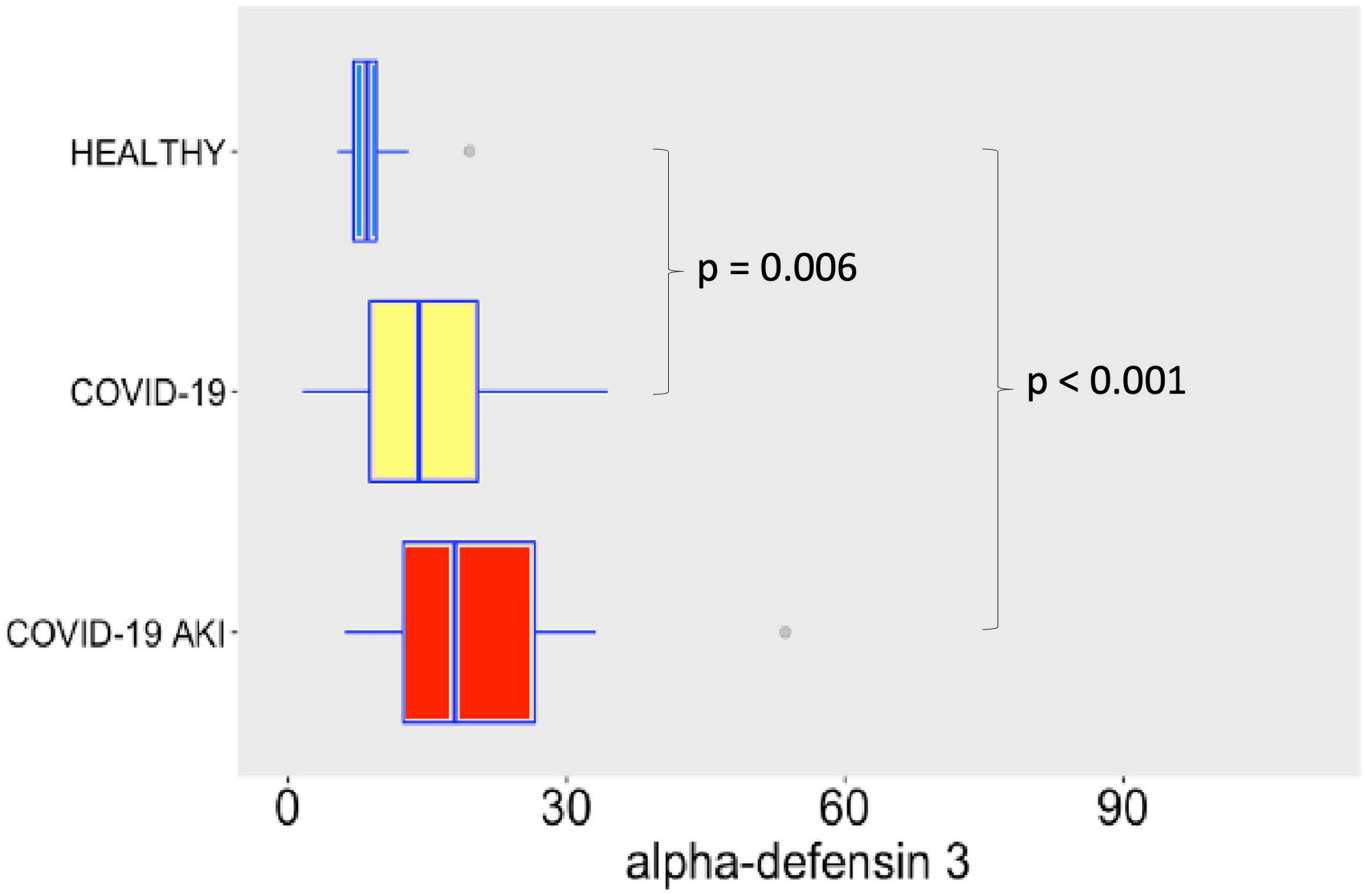
Plasma levels of α‐defensin 3 in ng/mL. The dots represent outliers.

**FIGURE 4:**
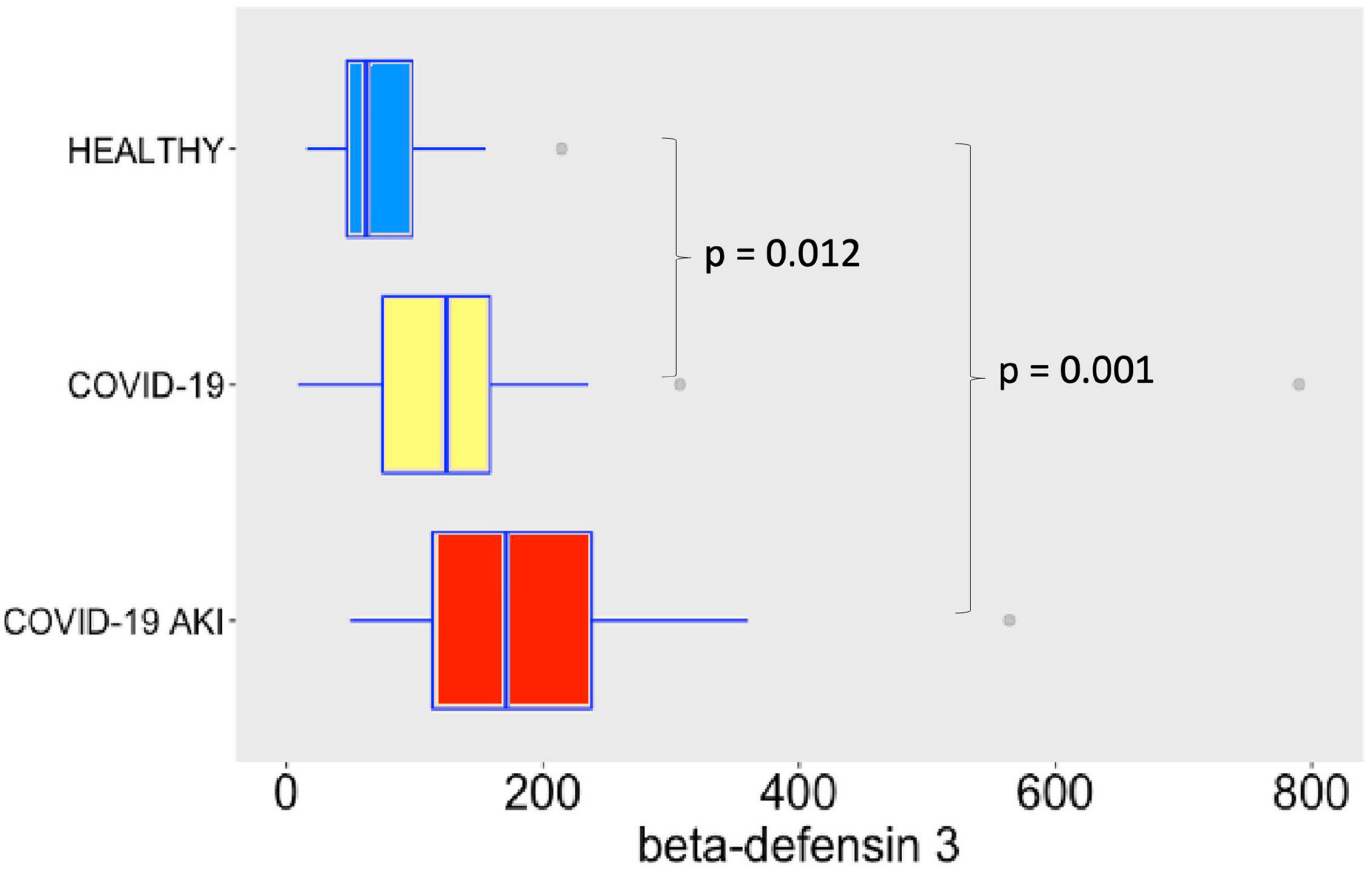
β‐defensin 3 serum levels in pg/mL. The dots represent outliers.

**FIGURE 5:**
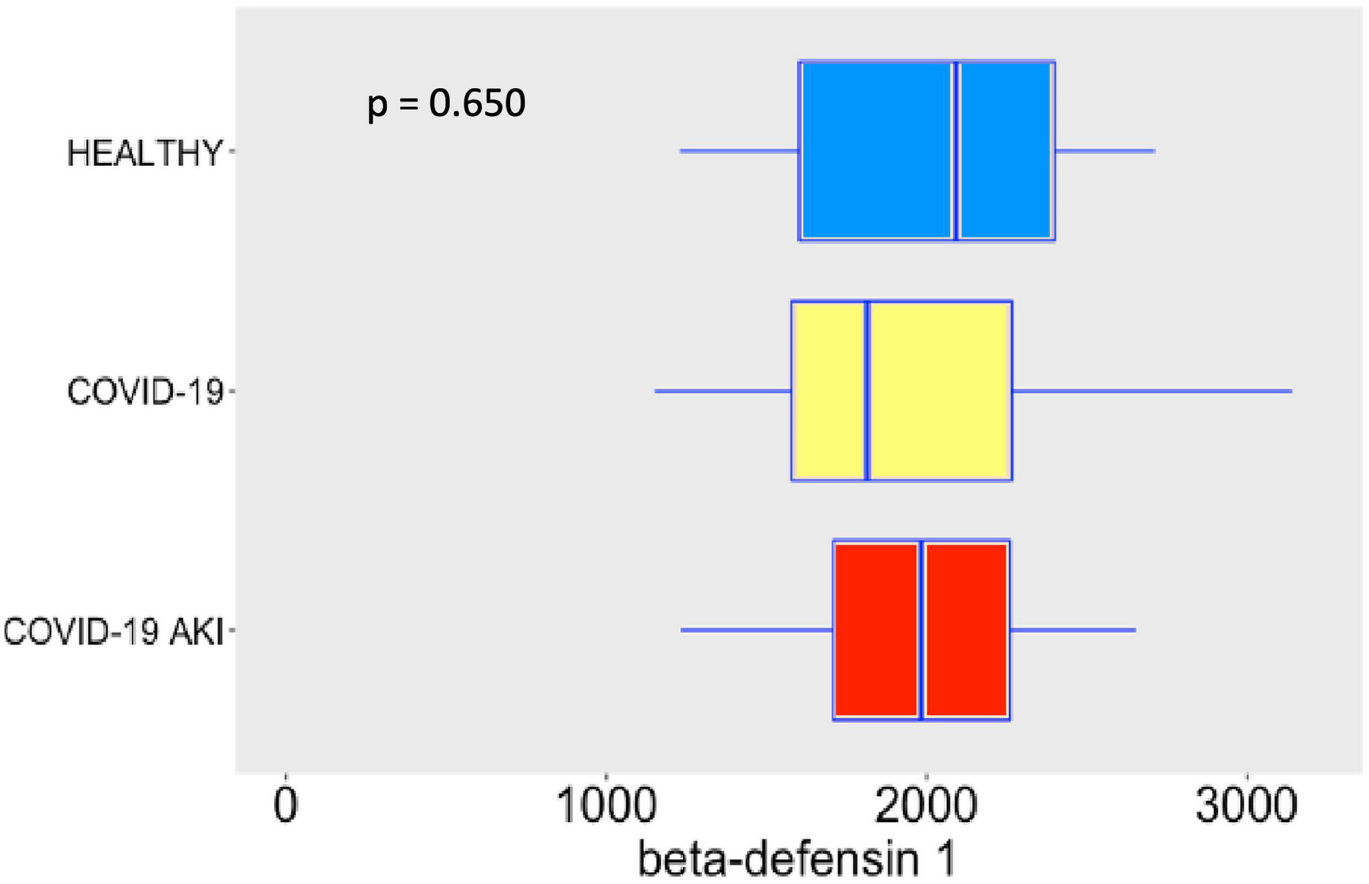
β‐defensin 1 plasma levels in pg/mL. The dots represent outliers.

Regarding AMPs and cytokines, SARS‐CoV‐2 infection induced robust release of α‐defensin 1 (Figure 2), especially in the AKI group, possible denoting a role to α‐defensin 1 in the pathogenesis of AKI. In the case of α‐defensin 3 (Figure 3) we observe a difference between COVID‐19 groups and the control group, but not between COVID‐19 groups. This difference may denote a role to α‐defensin 3 in the pathogenesis of COVID‐19. Although not significant, one can notice a tendency of increasing levels of α‐defensin 3 in COVID‐19 AKI group. Maybe a bigger sample would show statistically significant differences. β‐defensin 3 levels also significantly increased following SARS‐CoV‐2 infection (Figure 4). Other study already described β‐defensin 3 as a potential blocker to viral entry in human cells (Chen et al., 2018). There was no difference, however, in β‐defensin 1 plasma levels among the study groups (Figure 5).

**FIGURE 6:**
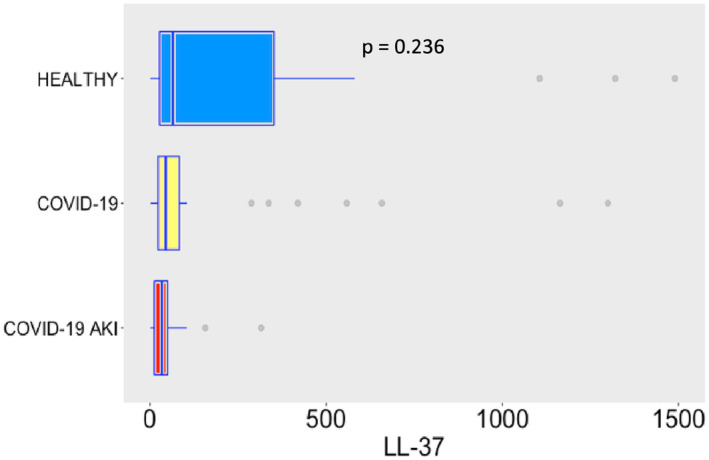
Plasma levels of LL‐37 in the study groups. LL‐37 values are in ng/mL. The dots represent outliers.

Plasma LL‐37 levels in our COVID‐19 patients were very similar to basal levels (control group), putting in evidence that the LL‐37 secretion system did not respond either (Figure 6). Importantly, a prior study from our group demonstrated inhibition of LL‐37 gene expression during septic shock, in comparison with patients in sepsis, while LL‐37 plasma levels in both sepsis and septic shock remained at similar levels than the healthy controls (Barbeiro et al., 2013).

**FIGURE 7:**
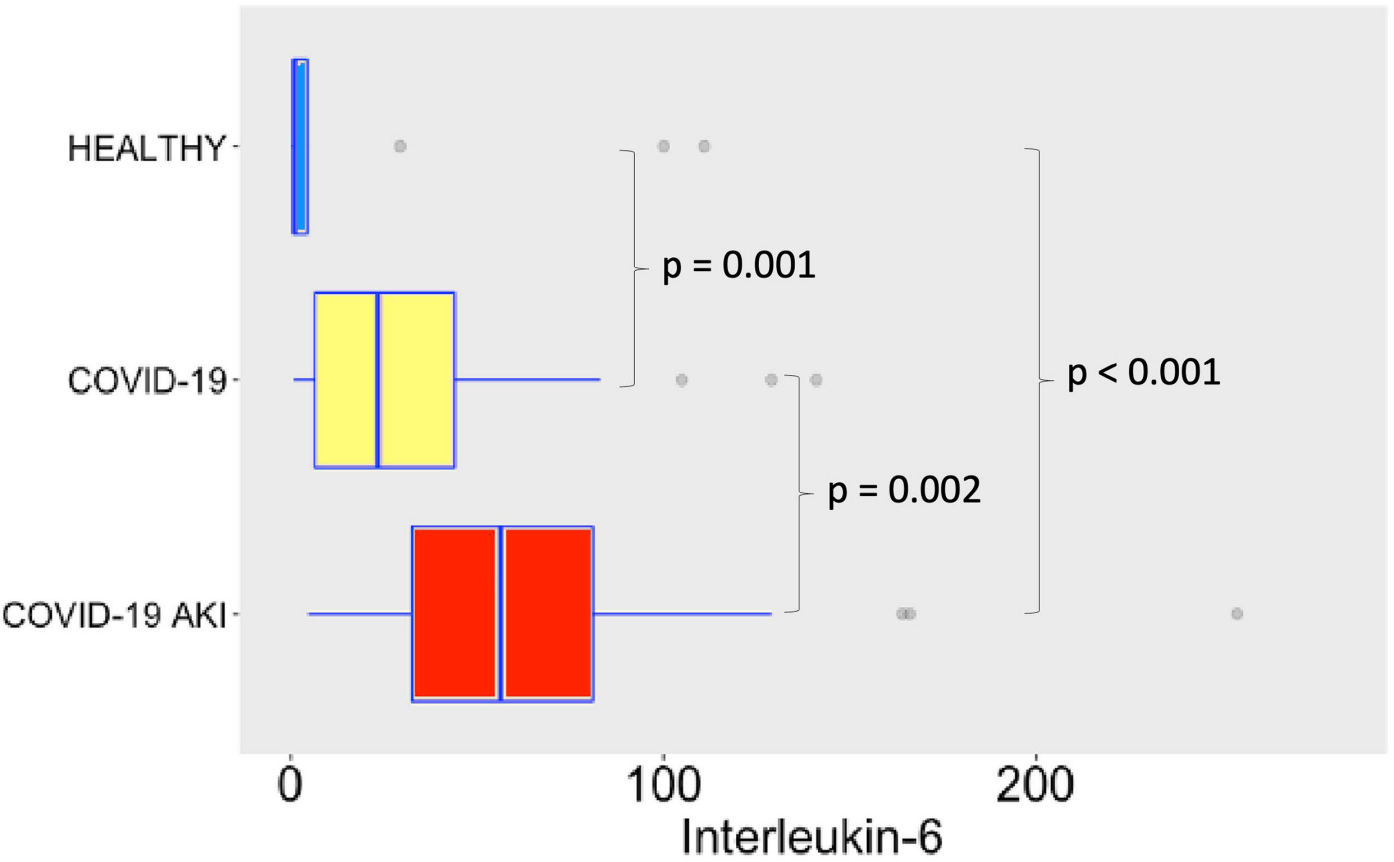
IL‐6 plasma levels in pg/mL. The dots represent outliers.

**FIGURE 8:**
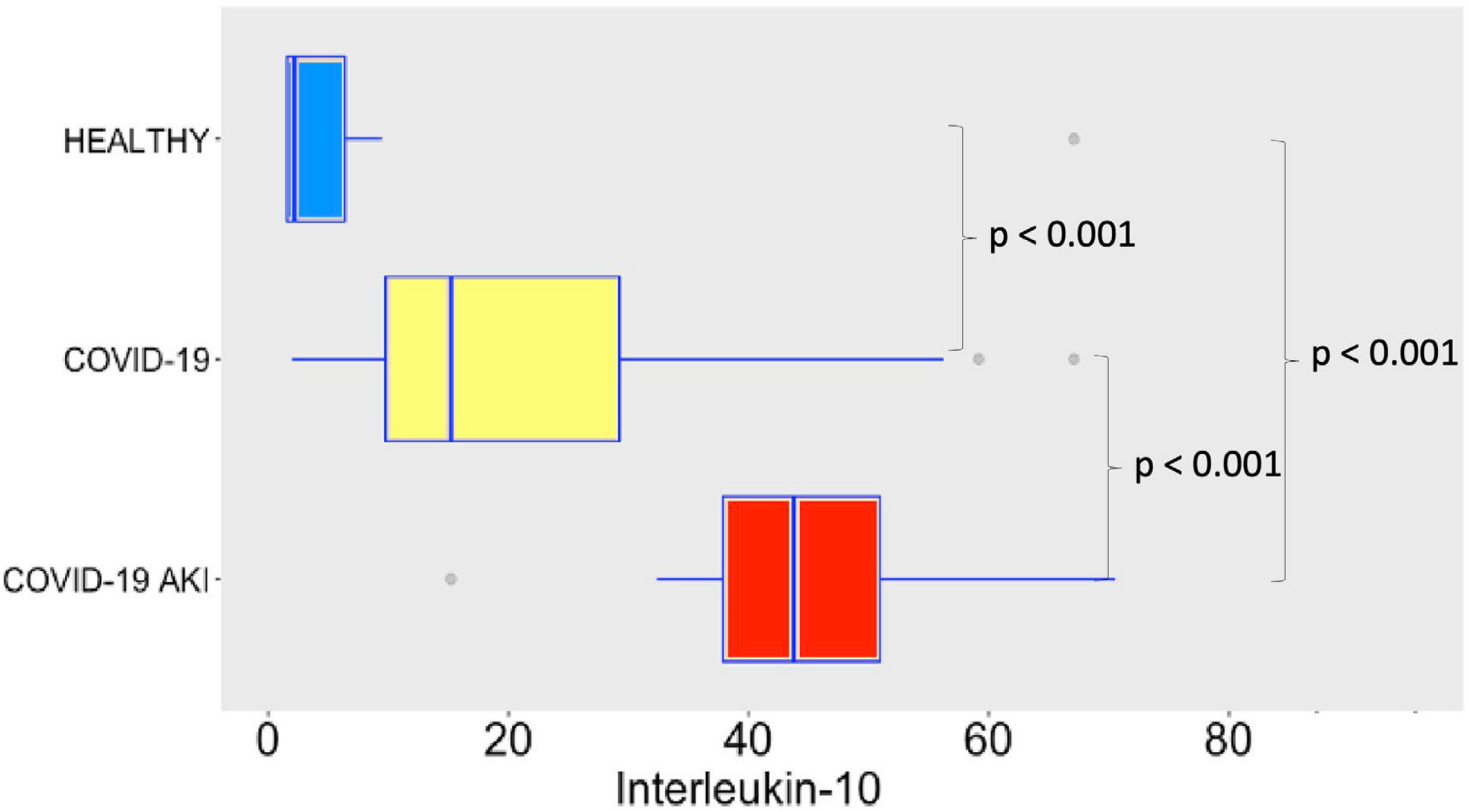
IL‐10 plasma levels in pg/mL. The dots represent outliers.

**FIGURE 9:**
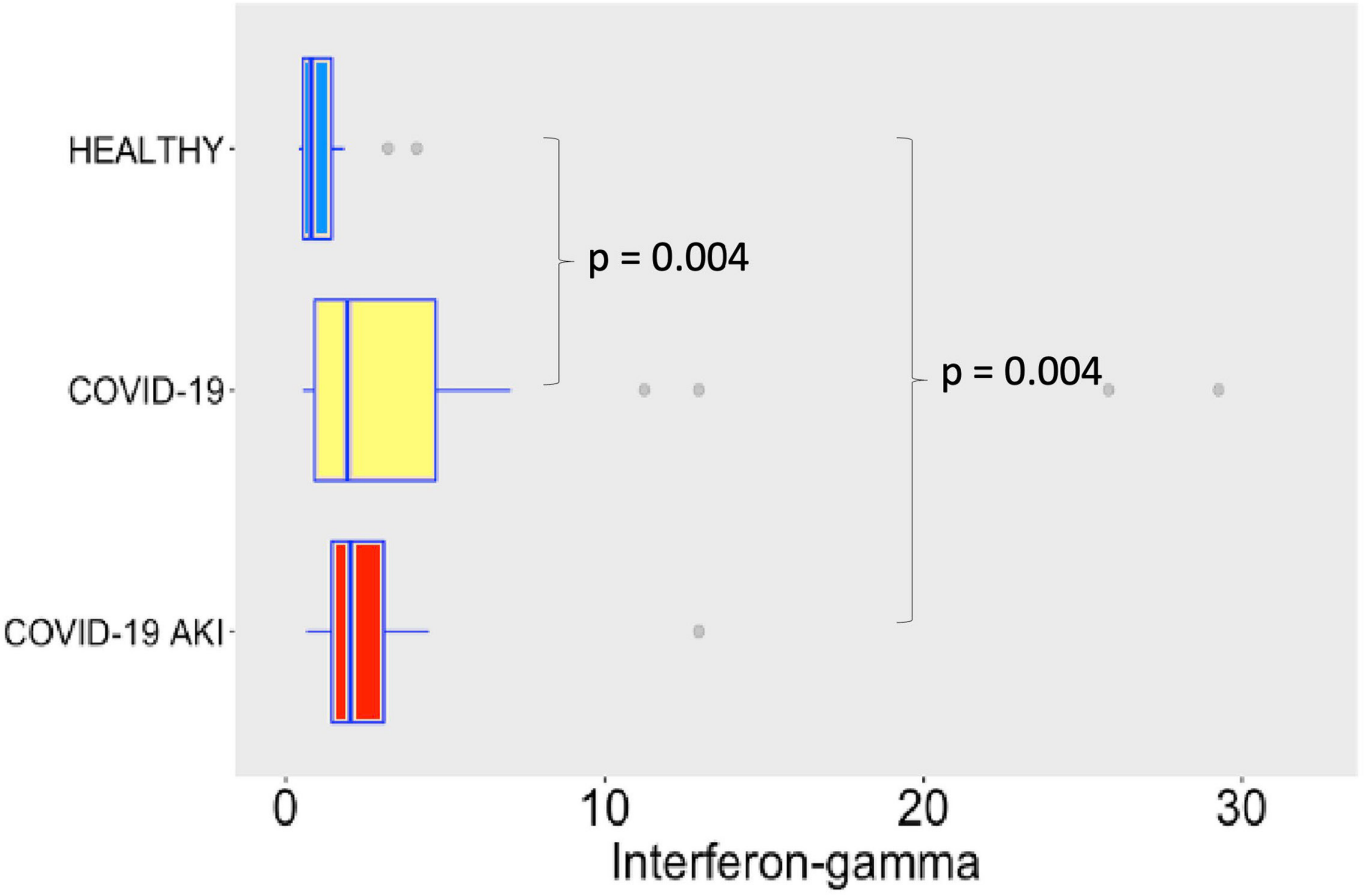
Interferon‐gamma plasma levels in pg/mL. The dots represent outliers.

**FIGURE 10:**
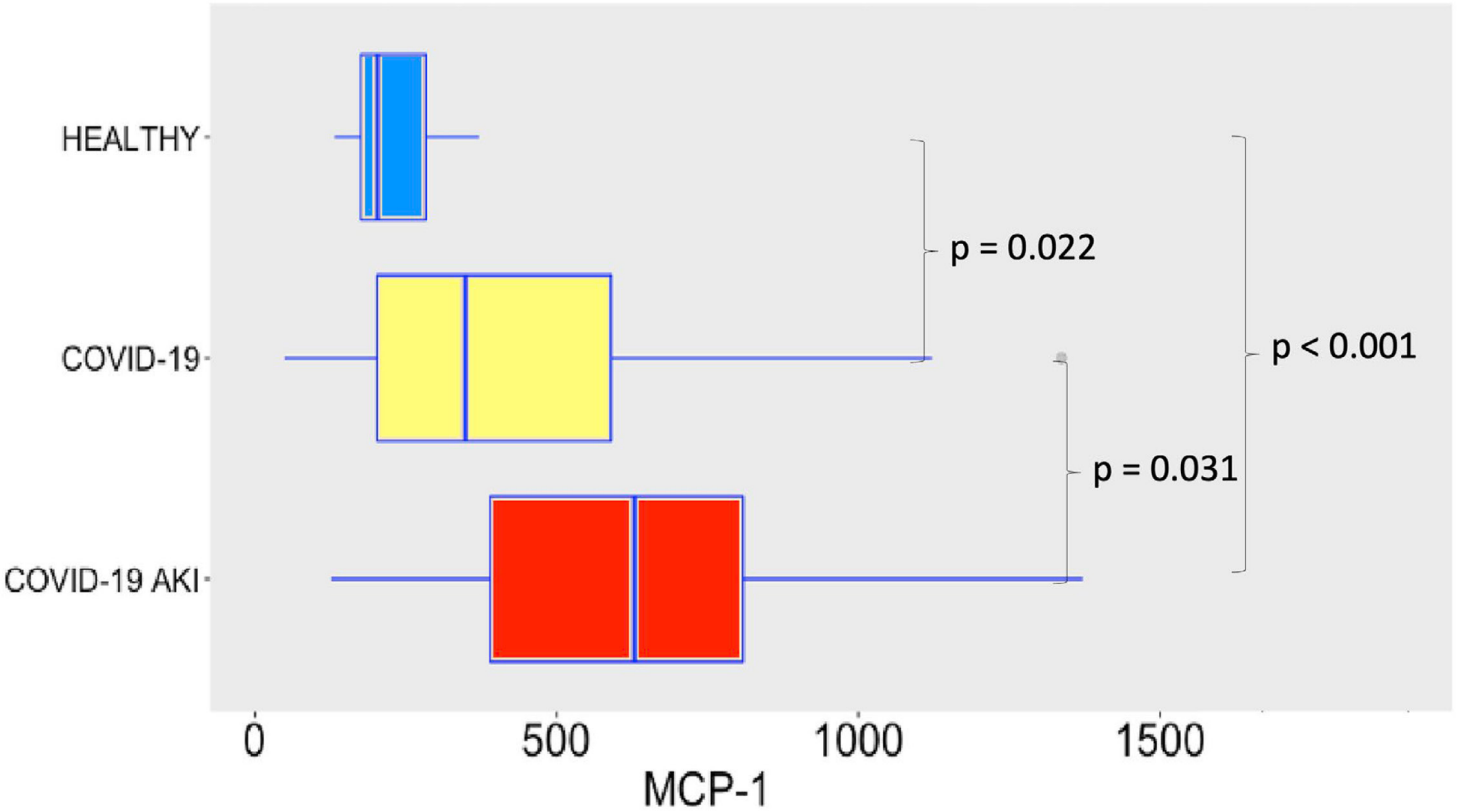
Plasma levels of MCP‐1 in pg/mL. The dots represent outliers.

**FIGURE 11:**
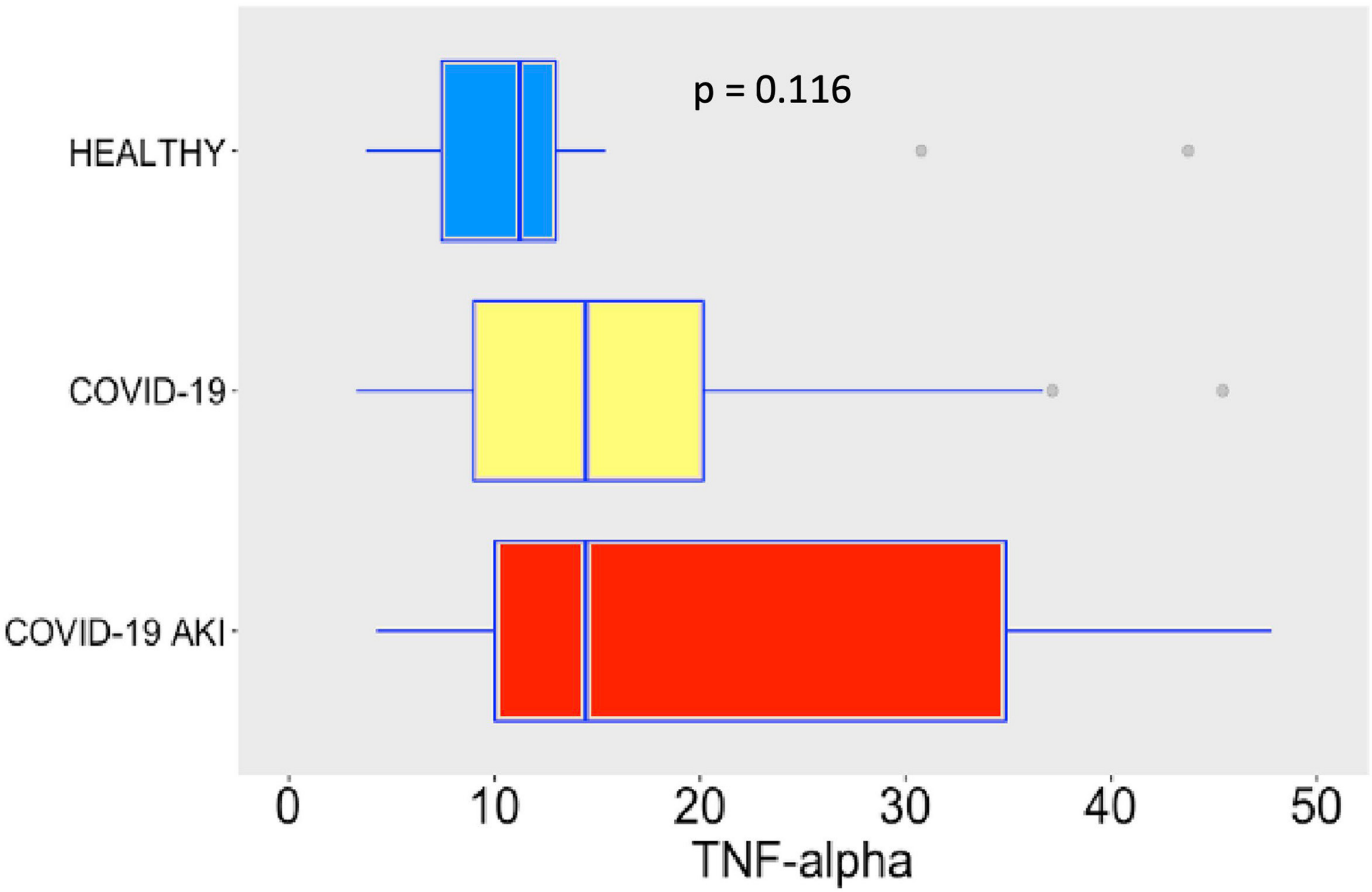
TNF‐alpha plasma levels, in pg/mL. The dots represent outliers.

**FIGURE 12:**
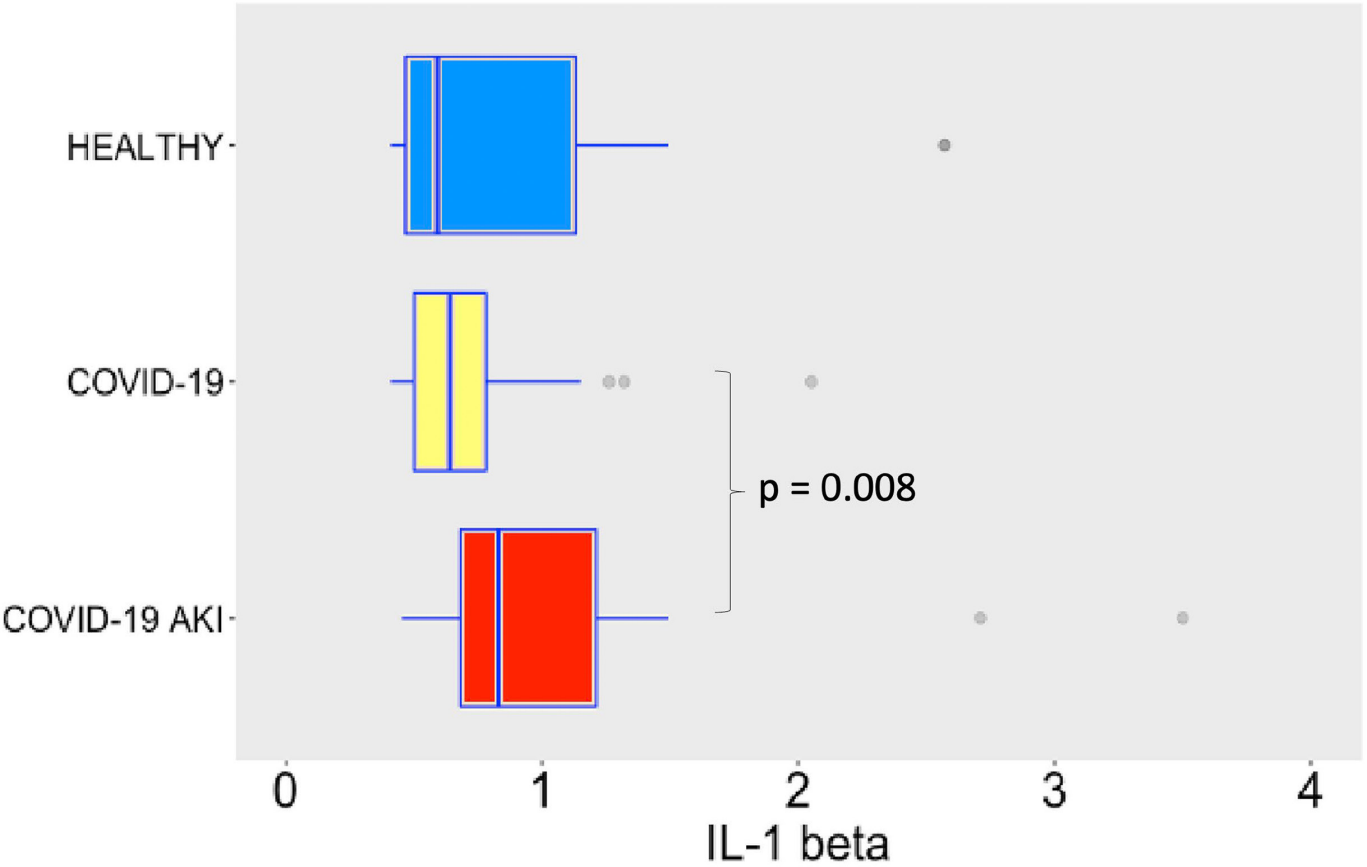
IL‐1 beta plasma levels in pg/mL. The dots represent outliers.

COVID‐19 induced potent secretion of IL‐6 (Figure 7), with greater levels of IL‐6 in AKI group. It does confirm a finding previously seen in other studies about COVID‐19, AKI and cytokine storm (Ahmadian et al., 2021). IL‐10 (Figure 8), IFN‐γ (Figure 9), and MCP‐1 (Figure 10) are also increased, even though no difference was observed between COVID‐19 and COVID‐19 AKI groups in what concerns INF‐gamma. It contrasts with the idea that cytokine storm may plays a role in AKI in COVID‐19 patients, because IFN‐gamma is a promoter of the cytokine storm pattern. There was no significant secretion of TNF‐α and IL‐1β in the COVID‐19 groups, when compared with the healthy controls (Figures 11 and 12), although we can observe a greater secretion of IL‐1B in patients with AKI, which may denote a role for this cytokine in the pathogenesis of this condition.

## 4 DISCUSSION

### 4.1 Laboratory routine

In our study, routine In‐hospital laboratory parameters proved to be poor indicators of disease severity in our COVID‐19 population. Even CRP levels were similar between groups. However, a recent study from our university also investigated several routine laboratory parameters in COVID‐19 patients and found interesting differences, when patients were grouped by gender or age (Ten‐Caten et al., 2021). For instance, markers such as CRP, ferritin, fibrinogen, LDH, and GGT were markedly elevated in many COVID‐19 patients in this study, particularly in older men, although similar profiles were observed between men and women once patients were admitted to the ICU. Additionally, complete blood count data revealed low platelet, basophil, and eosinophil counts in both male and female COVID‐19 patients. Thrombocytopenia and lymphopenia, considered predictors of disease severity in COVID‐19, displayed modest reductions compared to individuals without SARS‐CoV‐2 infection of similar age and sex. In our study, we also found high levels of CRP, although no difference between groups was observed. We also observed low eosinophils and lymphocytes count. We attribute the lack of significant differences in laboratory routine in between groups to the fact that our sample size is small, and we did not compare laboratory profiles for different genders, but between rather homogeneous samples with a predominance of older men in each group.

### 4.2 Antimicrobial peptides

SARS‐CoV‐2 induced robust release of α‐defensin 1, α‐defensin 3, and β‐defensin 3, but the LL‐37 and, surprisingly, β‐defensin 1 secretion systems were not activated. We anticipated a HBD‐1 response since it is expressed in kidney cells, but this was not the case. It seems that the main mechanism of kidney damage is related to macrophage activation and expression of α‐defensins. According to our findings, β‐defensin 3 is also implicated in this mechanism of disease. The role of defensins, however, cannot be oversimplified. Since defensins, in the same way as cathelicidins, can also exhibit both inflammatory and anti‐inflammatory properties (Fruitwala et al., 2019), further studies are necessary for a deeper comprehension of their activity, when SARS‐CoV‐2 spreads throughout the body.

### 4.3 Cytokines

While IL‐6, IL‐10, IFN‐γ and MCP‐1 plasma levels significantly increased following SARS‐CoV‐2 infection, the TNF‐α and IL‐1β pathways remained undisturbed. We believe that differences in TNF‐α and IL‐1β plasma levels could not be detected in the COVID‐19 groups, in comparison with the healthy individuals, because the cytokine storm is milder in SARS‐CoV‐2 infections than in bacterial sepsis (Dong et al., 2020), being more difficult to detect. The other cytokines, however, clearly indicate that systemic inflammation is a hallmark of severe COVID‐19 infections, occurs in association with the development of AKI. Indeed, higher levels of IL‐6 and IL‐10 have been described as predictors of more severe disease in COVID‐19 (Han et al., 2020). Moreover, a study with patients undergoing cardiac surgery showed that both IL‐6 and IL‐10 are related to AKI. It also showed that IL‐10 has a significant statistical correlation with levels of neutrophil gelatinase‐associated lipocalin, a well‐known biomarker of AKI (Zhang & Parikh, 2019). In COVID‐19 patients, elevated IL‐10 is a strong predictor of severe disease, as shown by a recent study (Henry et al., 2021; Udomsinprasert et al., 2021). The role of IL‐10 in the pathophysiology of AKI is not clearly understood but it seems that it has a protective role, at least in some AKI experimental models such as ischemia–reperfusion related AKI (Wei et al., 2022). In multiple experimental models of AKI, the role of IL1B as a promoter of inflammation and as a marker of worse renal outcome are described (Anders, 2016). In COVID‐19 patients IL1B has been perceived in bronchoalveolar lavage fluid and this cytokine seems to be related with the severe spectrum of this disease (Makaremi et al., 2022). Despite that, Medeiros et al. found a poor correlation between IL1B levels and SARS‐CoV2 viral load (Medeiros et al., 2022).

### 4.4 IL‐10 and the product IL‐10 × IL‐1B—A ROC curve and correlation analysis

The ROC curve, depicting test sensitivity against its false positive rate (FPR, which equals to 1 − specificity), is a powerful tool for assessing diagnostic test performance. Additionally, it aids in determining the best cut‐off value to ascertain the existence or non‐existence of a disease. The area under the ROC curve (AUC) is a widely used measure of diagnostic test accuracy. A higher AUC, closer to 1.0, indicates better test performance, seen when the ROC curve approaches the upper left corner, signifying perfect sensitivity and specificity. An AUC of 0.5, observed when the ROC curve aligns with the diagonal line (*y* = *x*), represents randomness, akin to a coin toss, rendering it ineffective as a diagnostic tool. For a meaningful diagnostic technique, the AUC must surpass 0.5 and preferably exceed 0.8 to be considered acceptable. An extensive review concerning the ROC curve analysis can be read elsewhere (Nahm, 2022).

**FIGURE 13:**
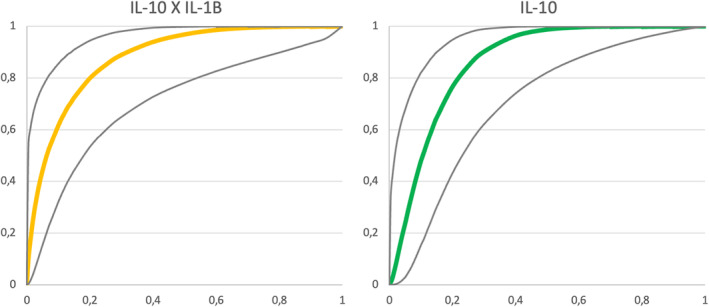
ROC Curves for IL‐10 and the product IL‐10 × IL‐1B to differentiate COVID‐19 with AKI from COVID‐19 patients. Gray lines delimitate the 95% Confidence Interval of the curves. *X*‐axis: 1 − specificity; *Y*‐axis: sensitivity.

As shown in Figure 13, both IL‐10 and the product IL‐10 × IL‐1B had a good performance in discriminate AKI from non‐AKI patients, according to the ROC curve analysis. The area under the curve (AUC) for IL‐10 was 0.86 (CI 0.77–0.94) and for the product IL‐10 × IL‐1B was 0.88 (CI 0.80–0.97), both with a *p*‐value < 0,001. The optimum ROC curve point (defined by Youden's index − sensitivity + specificity − 1) for IL‐10 (cut‐off 30.1 pg/mL) showed a sensitivity of 94% and a specificity of 75%, while the optimum ROC curve point for the product IL‐10 × IL‐1B (cut‐off 24.48 pg^2^/mL^2^) resulted in a sensitivity of 88% and a specificity of 83%. The Table 3 shows the AUC, sensitivity, specificity and cut‐off of another cytokines and AMPs as well. Although a causal relationship isn't possible to infer with our data, it's of notice that IL‐10 may be related to the pathophysiology of AKI in COVID patients (Pearson correlation coefficient between IL‐10 and the Highest Creatinine, a surrogate of AKI, was 0.508, *p*‐value < 0.001), a finding that contradicts the findings of another study with an experimental model of ischemia–reperfusion AKI in which IL‐10 showed potential of protective effect (Tang et al., 2020).

**TABLE 3:** ROC curve analysis of selected cytokines and AMPs to differentiate COVID‐19 with AKI from COVID‐19 patients.

Biomarker	AUC	95% CI	Cut‐off	Sens	Spec	*p*‐Value
IL‐6 (pg/mL)	0.76	0.65–0.87	30.4	82%	64%	0.002
IL‐10 (pg/mL)	0.86	0.77–0.94	30.1	94%	75%	<0.001
IL‐1B (pg/mL)	0.73	0.61–0.85	0.6	88%	44%	0.008
MCP‐1 (pg/mL)	0.68	0.56–0.81	386.0	76%	58%	0.031
DEFA1 (ng/mL)	0.70	0.57–0.83	3.33	71%	61%	0.018
DEFA3 (ng/mL)	0.67	0.53–0.80	14.16	71%	50%	0.052
IL‐10 × IL1B (pg^2^/mL^2^)	0.88	0.80–0.97	24.484	88%	83%	<0.001

## 5 CONCLUSION

The robust release of *α‐defensin 1 and α‐defensin 3* in patients with COVID‐19 and AKI in comparison with those with COVID‐19 alone is of interest because it may be a potential pathway to reduce the development of AKI. Moreover, given our findings, the role of IL‐10 in the pathophysiology of AKI must be further investigated so we have a better understanding of AKI in COVID‐19.

In the last few years, the struggle to find better biomarkers of AKI other than creatinine resulted in the description of many biomarkers, like KIM‐1, NGAL, TIMP‐2, and IGFBP7. Although none of them are perfect, the product TIMP‐2 × IGFBP7 has shown good performance in the Sapphire study (Kashani et al., 2013), with an AUC of 0.80. In our study, the product IL‐10 × IL‐1B showed an AUC of 0.88 to discriminate AKI. Of notice, our blood samples were collected in the first 24 h of hospitalization, before the detection of AKI, as defined according to creatinine criteria. Further studies are needed to define whether these biomarkers have such a good performance in a bigger and/or diverse population, so we can implement them in the clinical practice.

## AUTHOR CONTRIBUTIONS

FPS and HPS conceived the study. LFTS and HVB collected the samples. LFTS, HVB and DFB performed the experiments. LFTS and FPS performed the statistical analysis. FPS and HPS supervised the project. FPS and LFTS wrote the first manuscript. LFTS wrote the final manuscript.

## CONFLICT OF INTEREST STATEMENT

The authors declare no conflicts of interest.

## ETHICS STATEMENT

This study received approval from the Ethics Committee of our hospital (protocol # 30417520.0.0000.0068).
