# Correction to “Antimicrobial peptides and other potential biomarkers of critical illness in SARS‐CoV‐2 patients with acute kidney injury. AMPAKI‐CoV study”

**DOI:** 10.14814/phy2.16116

**Published:** 2024-06-23

[Theotonio Dos Santos LF, Barbeiro HV, Barbeiro DF, de Souza HP, Pinheiro da Silva F. Antimicrobial peptides and other potential biomarkers of critical illness in SARS‐CoV‐2 patients with acute kidney injury. AMPAKI‐CoV study. Physiol Rep. 2024 Feb;12(3):e15945. doi: 10.14814/phy2.15945. PMID: 38328863; PMCID: PMC10851028]

There is an error in the table 1 legend.

The original text is as follows: Mann–Whitney test was used to compare symptoms duration, days in mechanical ventilation, length of stay, rates of ICU admission, and mortality of the COVID groups. The Kruskal–Wallis test was used to analyze the other variables in the three study groups (IQR = interquartile range). Bold values indicate significant of *p*‐values.

The correct text is as follows: The Kruskal–Wallis test was used to compare ages between groups. The Mann–Whitney test was used to compare the duration of symptoms and length of stay in the COVID groups. The Chi‐square test was used to analyze the other variables between the two COVID‐19 groups and between the three study groups. (IQR = interquartile range).

We apologize for this error which does not affect the conclusions of the study.

